# Effects of Dexamethasone and Pentoxifylline on Mania-like and Depression-like Behaviors in Rats

**DOI:** 10.3390/ph15091063

**Published:** 2022-08-26

**Authors:** Ahmad Nassar, Abed N. Azab

**Affiliations:** 1 Department of Clinical Biochemistry and Pharmacology, Faculty of Health Sciences, Ben-Gurion University of the Negev, P.O. Box 653, Beer-Sheva 8410501, Israel; 2 Department of Nursing, Faculty of Health Sciences, Ben-Gurion University of the Negev, P.O. Box 653, Beer-Sheva 8410501, Israel

**Keywords:** brain, corticosteroid, cytokines, inflammation, mood disorders, tumor necrosis factor-α

## Abstract

Several studies support the notion that inflammation plays a role in the pathophysiology and treatment approaches of psychiatric illnesses, particularly mood disorders. Congruently, classic anti-inflammatory drugs were found efficacious in randomized clinical trials of patients with mood disorders. Moreover, accumulating data indicate that psychotropic drugs exhibit some anti-inflammatory effects. This study was undertaken to examine the efficacy of dexamethasone (a potent corticosteroid) and pentoxifylline (a methylxanthine drug with proven anti-tumor necrosis factor-α inhibitory activity) in behavioral models in rats, which were treated intraperitoneally with either dexamethasone or pentoxifylline for two weeks and then subjected to a battery of behavioral tests. Treatment with pentoxifylline, but not dexamethasone, was associated with antidepressant-like and anti-manic-like effects. The beneficial behavioral effects of pentoxifylline were accompanied by a prominent reduction in pro-inflammatory mediator levels in the brain. For the first time, the current work proves the efficacy of pentoxifylline against both mania-like and depressive-like behaviors. These results suggest that pentoxifylline may be a promising therapeutic intervention for patients with mood disorders. Taking into account the excellent tolerability profile of pentoxifylline in humans, it is warranted to conduct randomized clinical trials to investigate its therapeutic efficacy in patients with psychiatric disorders.

## 1. Introduction

Mental illnesses influence millions of people around the world, causing enormous anguish to and placing an intolerable burden on patients and their relatives. Mental illnesses are associated with high rates of death (due to increased suicidality and high incidence of medical ailments), increased comorbidity and chronic manifestation of symptoms [1,2]. The life-time prevalence of major psychiatric disorders is patently high among the general population; the prevalence of anxiety disorders, depressive disorders, bipolar disorder and schizophrenia are around 12%, 10%, 1.5% and 1%, respectively [3]. These data highlight the necessity of improving treatments for patients with mental disorders.

Irregular immune responses and altered levels of inflammatory mediators have been noticeably connected to the pathophysiology of depression [4,5,6,7], bipolar disorder [5,6,8,9] and schizophrenia [5,6,7], as well as other psychiatric illnesses [10]. In line with these findings, a large body of data suggests that inhibition of inflammation may contribute to the therapeutic dynamics of mood stabilizers, antidepressants and antipsychotic drugs [4,11,12,13].

Prostaglandins (PGs) are biologically active constituents that are produced by the cyclooxygenase (COX) enzymes. PGs modify many functions of the brain. For example, PGE2 regulates the hypothalamus–pituitary–adrenal (HPA) axis function, neurotransmitter release and synaptic transmission, thermoregulation and appetite [14]. Several works of research bolster the notion that PGs are involved in the pathophysiology of mental illnesses [15,16]. Most studies found that levels of PGs (particularly PGE2) are increased in plasma of patients with major depression [17], as well as those with other mental disorders [15]. Congruent with the aforementioned findings that PGE2 levels are increased in patients with mental disorders, numerous studies have demonstrated that drugs that inhibit the COX enzymes and reduce PGs synthesis exhibit beneficial effects in patients with mental disorders [18,19,20]. For example, Müller et al. observed in a randomized, double-blind clinical trial that addition of celecoxib—a selective COX-2 inhibitor—to amisulpride [20] assuaged symptoms and enhanced treatment response in patients with schizophrenia. Moreover, celecoxib add-on therapy evinced beneficial outcomes in double-blind, placebo-controlled trials of patients with major depression [19] and bipolar disorder [18]. Aspirin, a non-selective COX inhibitor, was determined therapeutically efficacious in patients with mood disorders [21]. In contrast to these positive findings, contradicting results have also been reported [22].

IL-6 is a pro-inflammatory cytokine that modulates various metabolic, regenerative and neuronal processes [23,24]. In the brain, IL-6 was found to influence the level and activity of neurotransmitters. Many studies demonstrated that IL-6 levels are elevated in patients with mental disorders, such as major depression [4,5,6], bipolar disorder [5] and schizophrenia [5,6]. On the other hand, some studies reported that IL-6 levels are unchanged or even decreased in psychiatric patients [25]. Consistent with these negative findings, a randomized, double-blind, placebo-controlled trial showed that add-on therapy with tocilizumab—a specific IL-6 receptor antagonist—did not influence behavioral outcomes in patients with schizophrenia [26].

TNF-α is a prominent pro-inflammatory mediator produced by different immune cells in the periphery and by glia cells (in particular) and neurons in the brain [27]. TNF-α regulates the expression of many genes that are important for neuronal function and survival [28] and alters neurotransmitter release and synaptic transmission [29]. Elevated levels of pro-inflammatory cytokines, such as TNF-α, IL-1β and IL-6, were associated with abated neurogenesis that effectuated memory impairment and anhedonia in rats [30]. Moreover, TNF-α was found to increase blood–brain barrier (BBB) permeability, which was associated with depressive behavior [31]. The increase in BBB permeability seems to facilitate infiltration of peripheral inflammatory mediators and immune cells to the brain, which leads to depressive symptoms and other behavioral abnormalities [32].

Numerous studies have demonstrated that TNF-α levels are elevated in patients with major depression [4,5], bipolar disorder [5,33] and schizophrenia [5,33]. Despite conflicting results, overall, most of the published data suggest that TNF-α levels are elevated in patients with major depression and other mental illnesses. To tackle this unclarity, several anti-TNF-α compounds have been tested as potential treatments for depression. Accumulating data show that anti-TNF-α therapy may possess antidepressant effects [34]. For example, Raison et al. [35] reported that infliximab—a monoclonal antibody against TNF-α—reduced depressive symptoms in some patients with treatment-resistant depression. Adalimumab, another monoclonal antibody against TNF-α, was also found to reduce depressive symptoms among patients with chronic physical illnesses [34]. Similar to infliximab and adalimumab, etanercept, a soluble TNF-α receptor that inhibits TNF-α activity, reduced depressive symptoms among patients with psoriasis [36]. However, in contrast to these positive results, other studies have cast doubt on the efficacy of selective TNF-α inhibition as a therapeutic strategy for depression [34,37].

The HPA axis controls vital functions in the human body, including regulation of immune responses and inflammation, metabolic and cardiovascular physiology, cognitive function and mental health. It is well-established that corticosteroids confer potent anti-inflammatory effects [38]. Several publications have associated alterations in glucocorticoids levels and function with mood disorders [39].

Several clinical trials investigated the efficacy of corticosteroids as a treatment for psychiatric illnesses, revealing opposing results. Some studies of patients with depression delineated a significant reduction in depressive symptoms [40,41]. For example, Arana et al. [40] showed that treatment with oral dexamethasone resulted in a prominent reduction in depressive symptoms in patients with depression. In contrast to these reinforcing findings, some studies demonstrated that long-term use of corticosteroids may induce both depression and mania [42,43]. For example, Brown et al. [42] reported that chronic treatment with prednisone increased symptoms of mania and had no prominent effect on depression in asthmatic patients. Moreover, treatment with anti-glucocorticoid compounds demonstrated beneficial effects in patients with major depression [41]. Together, these findings complicate the interpretation of the summated results regarding the effectiveness of corticosteroid as a treatment for mood disorders.

Pentoxifylline (PTF) is a methylxanthine derivative that has been used for many years for the treatment of various clinical conditions [44,45,46,47]. Similar to other methylxanthines (e.g., theophylline and caffeine), PTF inhibits the enzyme phosphodiesterase [48]. Among the most well-established pharmacological properties of PTF is its TNF-α inhibitory effect [45,48,49,50,51,52]. PTF was shown to inhibit TNF-α synthesis under various experimental conditions both in vitro and in vivo (in animals and humans) [45,49,50,51,52], pointing to a potent anti-inflammatory effect. Due to the strong association between TNF-α and depression, the antidepressant-like effect of PTF was tested in a number of animal behavioral studies [51,52,53], revealing positive therapeutic effects. For example, in a study by Elgarf et al. [52], rats were repeatedly exposed to an inflammatory stimulus (lipopolysaccharide (LPS)) followed by a chronic mild stress (CMS) to induce depressive-like behavior. The combined exposure to LPS and CMS resulted in a significant increase in immobility time in the forced swim test (FST) [52]. In the FST, immobility time represents despair and depressive-like behavior [54,55]. Treatment with PTF or the antidepressant medication imipramine resulted in a profound decrease in immobility time, suggestive of a potent antidepressant-like effect of the drugs [52]. Moreover, Bah et al. [53] demonstrated that PTF exerted antidepressant-like effects in rats that were subjected to an experimental model of myocardial infarction. PTF significantly increased sucrose preference and significantly decreased immobility time in post-infarction rats [53]. The sucrose preference test is used to assess anhedonia [54,55], which is a behavioral feature of depression. Mohamed et al. [51] also observed that treatment with PTF 50 mg/kg (ip) for 3 weeks significantly increased sucrose preference in rats that were subjected to CMS. Consistent with the positive results in animal studies, El-Haggar et al. [56] have shown that PTF demonstrated beneficial effects as an add-on treatment in patients with major depression. Other randomized clinical trials also showed beneficial effects of PTF as a treatment for depression [57].

In the present study we aimed to uncover whether PTF confers other beneficial behavioral properties in addition to its antidepressant-like effect and, thus, we examined its effects on amphetamine (AMPH)-induced hyperactivity (a model for mania-like behavior) and aggressive behavior. Hyperactivity and aggressiveness are features that characterize some patients with bipolar disorder and psychotic disorders.

Dexamethasone (DEX) is a potent corticosteroid with profound anti-inflammatory properties, among other therapeutic traits. DEX is used in numerous inflammatory and immune-associated conditions [38]. The powerful pharmacological effects of DEX are associated with several adverse reactions, especially when the drug is given chronically and at high doses [58]. As mentioned above, DEX has been found to exhibit antidepressant effects in patients with major depression [40]. However, as far as we know, the efficacy of corticosteroids in general, and DEX in particular, has not been directly tested as a treatment for mania in a randomized, double-blind trial in humans.

As mentioned above, both PTF [56,57] and DEX [40] have been shown to exert potent antidepressant effects in human subjects, which may derive, at least in part, from their potent anti-inflammatory effects [38,45,48,49,50,51,52]. However, to the best of our knowledge, their anti-manic and anti-aggressive effects have never been tested directly in humans or in animals. Therefore, the present study was undertaken to examine the efficacy of PTF and DEX in rat models of depression, mania and aggression. Additionally, we tested their influence on levels of IL-6, PGE2 and TNF-α in rat brain.

## 2. Results

### 2.1. Effects of DEX and PTF on Immobility Time

**Figure 1:**
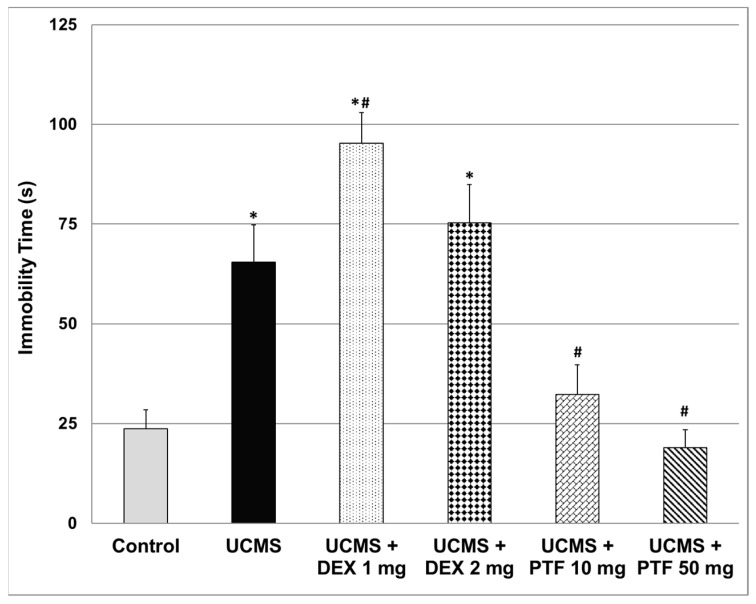
Effects of DEX and PTF on immobility time. Values are mean ± SEM of 11–12 rats in each group. One-way ANOVA test: F = 23.5, *p* < 0.0001; *t*-test: * *p* < 0.05 vs. control, ^#^ *p* < 0.05 vs. UCMS. DEX—dexamethasone, PTF—pentoxifylline, UCMS—unpredictable chronic mild stress.

At the end of an unpredictable chronic mild stress (UCMS) protocol and anti-inflammatory treatment, rats were subjected to a forced swim test (FST). As seen in Figure 1, UCMS rats had a significantly longer immobility time than control animals, suggestive of depressive-like behavior. Treatment with DEX 1 mg/kg significantly increased the (elongated) immobility time in UCMS rats, indicative of a depressive-like effect. On the other hand, PTF (10 and 50 mg/kg) significantly shortened immobility time (Figure 1), indicative of a potent antidepressant-like effect.

### 2.2. Effects of DEX and PTF on AMPH-Induced Hyperactivity

**Figure 2:**
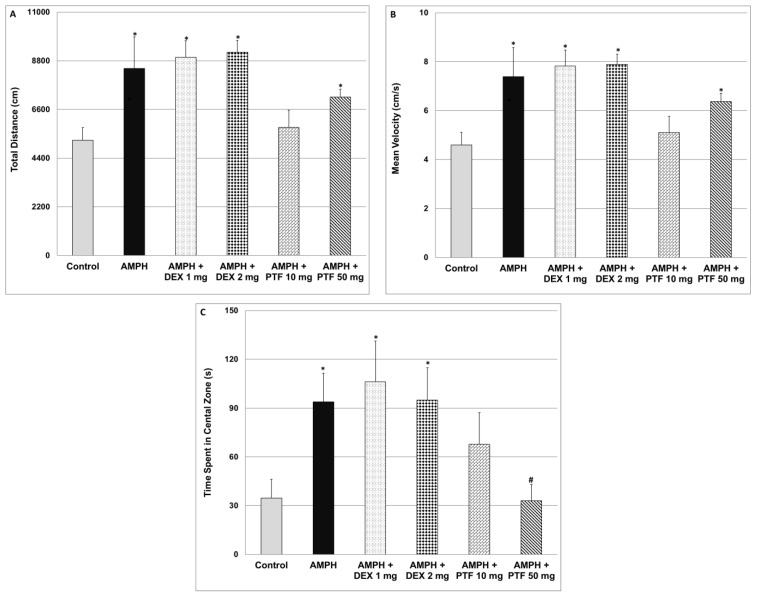
Effects of DEX and PTF on amphetamine-induced hyperactivity and risk-taking behavior. Values are mean ± SEM of 10–12 rats in each group. (**A**) One-way ANOVA test: F = 8.57, *p* < 0.0001; *t*-test: * *p* < 0.05 vs. control. (**B**) One-way ANOVA test: F = 8.05, *p* < 0.0001; *t*-test (two-tailed): * *p* < 0.05 vs. control. (**C**) One-way ANOVA test: F = 2.99, *p* = 0.0173; *t*-test (two-tailed): * *p* < 0.05 vs. control, ^#^ *p* < 0.05 vs. amphetamine. AMPH—amphetamine, DEX—dexamethasone, PTF—pentoxifylline.

As seen in Figure 2, administration of AMPH significantly increased total distance traveled (A) and (scalar) mean velocity (B). Treatment with DEX at both doses did not affect AMPH-induced elevation in these parameters. On the other hand, treatment with PTF 10 mg/kg significantly decreased the elevation in total distance (Figure 2A) and scalar mean velocity (Figure 2B), while PTF 50 mg/kg did not have a significant effect. Furthermore, AMPH significantly increased the time spent in the central zone of the open field (Figure 2C), indicative of increased risk-taking behavior. Treatment with DEX at both doses did not alter this parameter. In contrast, administration of PTF 50 mg/kg (but not 10 mg/kg) significantly decreased the time spent in the central zone (Figure 2C), suggestive of an anti-manic-like effect from PTF.

### 2.3. Effects of DEX and PTF on Aggressive-like Behavior

**Figure 3:**
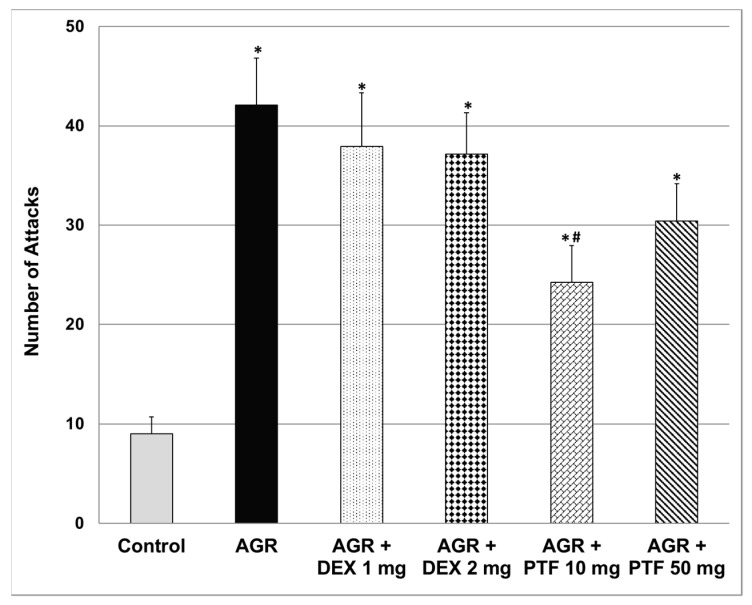
Effects of DEX and PTF on aggressive behavior. Values are means ± SEM of 11–12 rats in each group. One-way ANOVA test: F = 8.89, *p* < 0.0001; *t*-test: * *p* < 0.05 vs. control, ^#^ *p* < 0.05 vs. aggressive rats. AGR—aggressive, DEX—dexamethasone, PTF—pentoxifylline, RIAT—resident-intruder aggression test.

Aggressive (resident) rats executed a significantly higher number of attacks as compared to control animals (Figure 3). Pre-treatment with DEX (1 or 2 mg/kg) did not alter the aggressive behavior of resident rats. Contrastingly, 10 mg/kg PTF (but not 50 mg/kg) significantly decreased the number of attacks committed by resident rats (Figure 3).

### 2.4. Effects of DEX and PTF on TNF-α Levels in Brains of UCMS Rats

**Figure 4:**
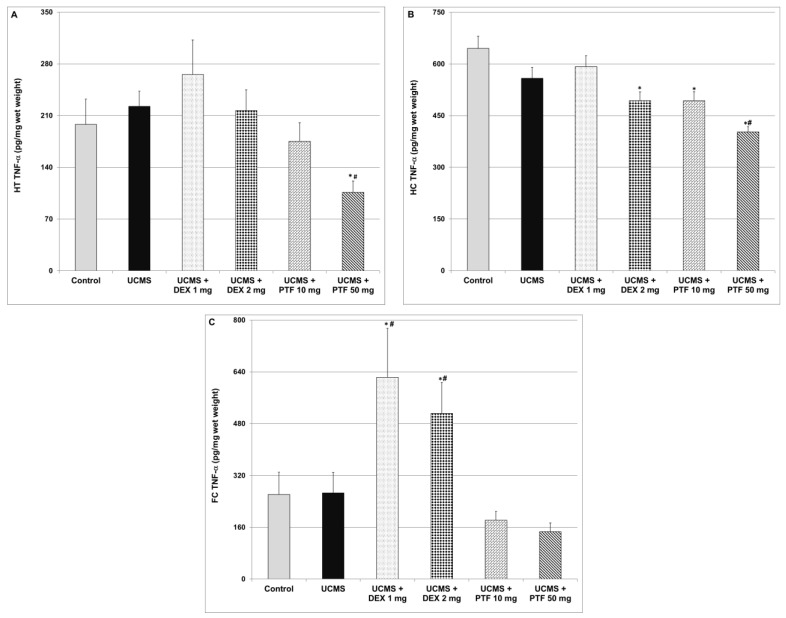
Effects of DEX and PTF on TNF-α levels in brains of UCMS rats. (**A**) Hypothalamus (HT), (**B**) hippocampus (HC) and (**C**) frontal cortex (FC). Values are means ± SEM of 11–12 rats in each group. (**A**) One-way ANOVA test: F = 3.19, *p* = 0.012; *t*-test: * *p* < 0.05 vs. control, ^#^ *p* < 0.05 vs. UCMS. (**B**) One-way ANOVA test: F = 11.82, *p* = 0.0001; *t*-test: * *p* < 0.05 vs. control, ^#^*p* < 0.05 vs. UCMS. (**C**) One-way ANOVA test: F = 6.09, *p* = 0.0004; *t*-test: * *p* < 0.05 vs. control, ^#^ *p* < 0.05 vs. UCMS. DEX—dexamethasone, PTF—pentoxifylline, TNF—tumor necrosis factor, UCMS—unpredictable chronic mild stress.

As seen in Figure 4, exposure to the UCMS protocol was not associated with significant changes in TNF-α levels in the three tested brain regions (HT, HC and FC). As compared to control and UCMS (only)-subjected rats, treatment with 50 mg/kg PTF was associated with a significant decrease in TNF-α levels in the HT (Figure 4A) and HC (Figure 4B) of UCMS rats. Treatments with 2 mg/kg DEX and 50 mg/kg PTF significantly decreased TNF-α levels in the HC (Figure 4B). In contrast, DEX (1 and 2 mg/kg) significantly increased TNF-α levels in the FC (Figure 4C).

### 2.5. Effects of DEX and PTF on IL-6 Levels in Brain of UCMS Rats

**Figure 5:**
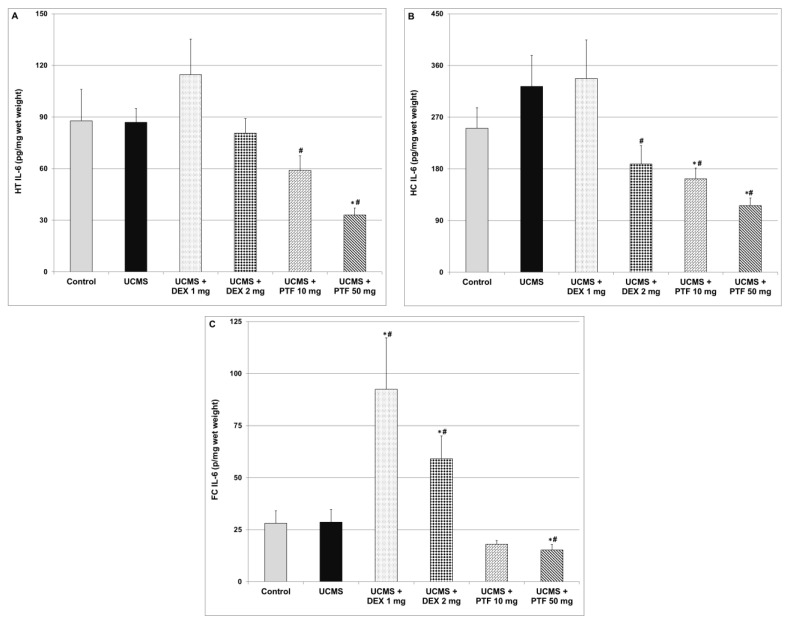
Effects of DEX and PTF on IL-6 levels in brains of UCMS rats. (**A**) Hypothalamus (HT), (**B**) hippocampus (HC) and (**C**) frontal cortex (FC). Values are means ± SEM of 11–12 rats in each group. (**A**) One-way ANOVA test: F = 5.11, *p* = 0.0014; *t*-test: * *p* < 0.05 vs. control, ^#^ *p* < 0.05 vs. UCMS. (**B**) One-way ANOVA test: F = 4.89, *p* = 0.0019; *t*-test: * *p* < 0.05 vs. control, ^#^ *p* < 0.05 vs. UCMS. (**C**) One-way ANOVA test: F = 7.44, *p* = 0.0001; *t*-test: * *p* < 0.05 vs. control, ^#^ *p* < 0.05 vs. UCMS. DEX—dexamethasone, IL—interleukin, PTF—pentoxifylline, TNF—tumor necrosis factor, UCMS—unpredictable chronic mild stress.

Similar to TNF-α, exposure to UCMS did not cause significant changes in IL-6 levels in the HT, HC and FC (Figure 5). As compared to UCMS (only)-subjected rats, treatment with 10 or 50 mg/kg PTF was associated with a significant decrease in IL-6 levels in the HT (Figure 5A) and HC (Figure 5B). Treatment with 50 mg/kg PTF also significantly reduced IL-6 levels in the FC (Figure 5C). Similarly, 2 mg/kg DEX significantly decreased IL-6 levels in the HC (Figure 5B). In contrast, DEX (1 and 2 mg/kg) significantly increased IL-6 levels in the FC (Figure 5C).

### 2.6. Effects of DEX and PTF on PGE2 Levels in Brains of UCMS Rats

**Figure 6:**
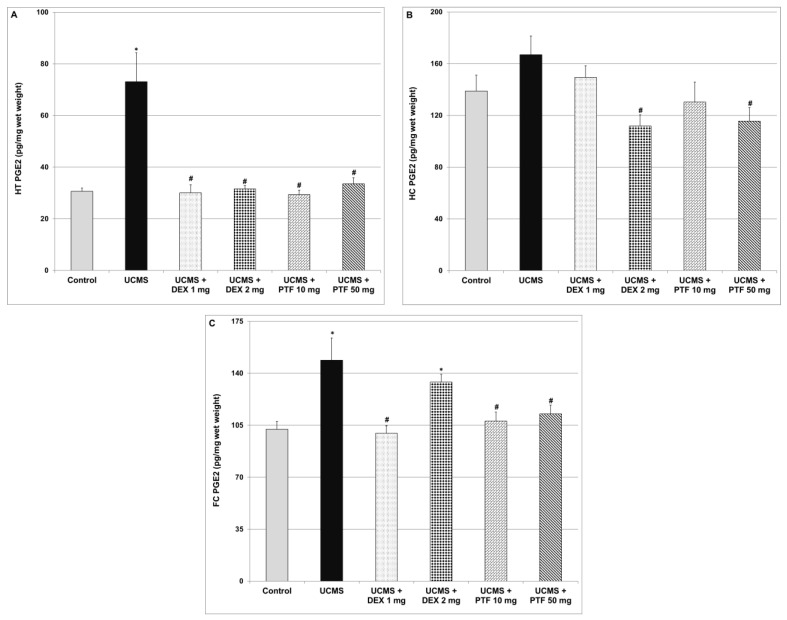
Effects of DEX and PTF on PGE2 levels in brains of UCMS rats. (**A**) Hypothalamus (HT), (**B**) hippocampus (HC) and (**C**) frontal cortex (FC). Values are means ± SEM of 11–12 rats in each group. (**A**) One-way ANOVA test: F = 12.85, *p* = 0.0001; *t*-test: * *p* < 0.05 vs. control, ^#^ *p* < 0.05 vs. UCMS. (**B**) One-way ANOVA test: F = 3.73, *p* = 0.0094; *t*-test: ^#^ *p* < 0.05 vs. UCMS. (**C**) One-way ANOVA test: F = 5.99, *p* = 0.0005; *t*-test: * *p* < 0.05 vs. control, ^#^ *p* < 0.05 vs. UCMS. DEX—dexamethasone, PG—prostaglandin, PTF—pentoxifylline, UCMS—unpredictable chronic mild stress.

Levels of PGE2 were significantly higher in the HT and FC of UCMS rats (Figure 6A and Figure 6C respectively). As compared to UCMS (only) rats, DEX and PTF treatments significantly decreased PGE2 levels in the HT of UCMS-subjected rats (Figure 6A). Treatments with 2 mg DEX and 50 mg/kg PTF also reduced PGE2 levels in the HC (Figure 6B). Moreover, 1 mg/kg DEX and PTF (10 and 50 mg/kg) led to significant decreases in PGE2 levels in the FC (Figure 6C).

## 3. Discussion

The major findings of the present study are: (1) PTF reduced immobility time in the FST, attesting to an antidepressant-like effect. Treatment with PTF was accompanied with a significant reduction in IL-6, PGE2 and TNF-α levels in the brain. (2) In contrast to PTF, treatment with DEX was associated with a pro-depressive-like effect. A significant increase in IL-6 and TNF-α levels in the FC may have contributed at least partially to this feature. (3) PTF attenuated the elevation in total distance travelled, velocity and time spent in the central zone in the AMPH-induced hyperactivity test. (4) PTF decreased the aggressiveness of animals in the RIAT model. These results imply that PTF may be a promising therapeutic option for patients with psychiatric disorders.

PTF has various pharmacological properties and is indicated for treatment of several illnesses [44,45,46,47]. Importantly, in adults, PTF was found to be safe and presented an excellent tolerability profile [42,45,47]. Moreover, PTF is BBB-penetrable, enabling high concentrations of the drug to reach the brain and induce neurological effects [45,49,50]. However, despite its recognized excellent tolerability profile, the multiple pharmacological effects of PTF may still be viewed as a two-edged sword and, in certain circumstances (e.g., when it is administered at especially high doses), may lead to adverse effects, such as gastrointestinal symptoms.

The primary objective of the present study was to examine whether PTF, adjunctive to its ostensible antidepressant effects [51,52,53,56], possesses *additional* behavioral therapeutic effects. Based on the large body of data suggesting that elevated TNF-α levels are associated with increased risk for mood disorders (among other psychiatric illnesses), we hypothesized that the potent anti-TNF-α effect of PTF would be associated with a prominent antidepressant-like effect. Consistent with previous findings [51,52,53,56], we observed that PTF exerted a potent antidepressant-like effect in rats that were subjected to a “depression-inducing” protocol: the UCMS (Figure 1). This antidepressant-like effect of PTF may derive, at least in part, from its inhibitory effects on pro-inflammatory mediator production in the brain, as it markedly decreased the levels of IL-6, PGE2 and TNF-α levels in the HT, HC and FC (Figure 4, Figure 5 and Figure 6). As mentioned, an important advantage of PTF as a potential treatment for psychiatric disorders is its BBB penetration ability, which enhances its effects on the brain. In contrast, specific TNF-α antagonists, which are large molecules and thus cannot cross the BBB, do not confer this kinetic property [34,59]. Notwithstanding their lack of BBB penetrance, these compounds were shown to exert beneficial brain-associated effects [35,36]. This is probably due to their inhibition of TNF-α activity in the periphery, which may also mitigate the inflammatory response in the brain. In line with this assumption, in a preliminary experiment in our lab, we observed that PTF (50 mg/kg, ip) profoundly decreased plasma TNF-α levels in lipopolysaccharide-treated rats (data not shown). Thus, the anti-TNF-α effect of PTF is prominent both in the periphery and in the brain. However, the antidepressant-like effect of PTF may also derive from other non-TNF-α-related effects. Consistent with the anti-depressive potential of PTF, a placebo-controlled, double-blind study found that addition of PTF (400 mg/day) to escitalopram (20 mg/day) for 12 weeks led to a significant improvement in depressive symptoms in adults with major depression [56]. The addition of PTF to escitalopram was associated with favorable biochemical changes, including a significant reduction in plasma TNF-α and IL-6 levels and an increase in plasma serotonin and brain-derived neurotrophic factor levels [56]. Recent randomized, double-blind clinical trials also showed that PTF exerts beneficial effects in patients with major depression [57].

To the best our knowledge, the present study is the first to show that PTF exhibits anti-manic-like effects. As seen in Figure 2, PTF prevented the AMPH-induced increase in rats’ locomotor activity. Hyperactivity and restlessness are prominent features in some manic and psychotic patients. Moreover, PTF significantly attenuated the increase in time spent in the central zone (of the open field) in AMPH-treated animals (Figure 2). Rats are naturally oriented to hide in closed and dark places during light time in order to avoid being observed by carnivores. Hence, staying in the peripheral zone of the arena (next to the walls) is deemed characteristic normal behavior of rodents. In contrast, if the time spent in the central zone significantly exceeds that detected in control rats, it is recognized as risk-taking behavior, which is another facet of manic behavior. Therefore, the reduction in time spent in the central zone by PTF may be interpreted as an anti-manic-like effect. PTF also significantly decreased the aggressiveness of rats in the RIAT model (Figure 3), attesting to another possible therapeutic benefit of this medication in manic/psychotic patients. Based on the facts that PTF has an excellent tolerability profile in humans and that it exhibited promising beneficial behavioral effects in animals, as well as in a clinical trial of patients with depression [56], it is necessary to investigate its therapeutic potential in other randomized clinical trials with patients with psychiatric disorders. In this regard, we are currently conducting a randomized, double-blind trial of adjunctive PTF treatment in patients with chronic schizophrenia (Israeli Ministry of Health trial registration number: MOH-2020-01-19-008647). A similar trial is currently being performed in Egypt (ClinicalTrials.gov identifier: NCT04094207). Moreover, another on-going single-arm, open-label trial (ClinicalTrials.gov identifier: NCT04417049) is being executed to test the efficacy of add-on PTF treatment in patients with acute bipolar depression. Hopefully, these trials will shed light on the therapeutic potential of PTF as a treatment for mental disorders.

In contrast to PTF, treatment with DEX effectuated a pro-depressant-like effect (Figure 1). We speculate that this result derives, at least in part, from the increase in IL-6 and TNF-α levels in the FC (Figure 4C and Figure 5C). Consistently, Shelton et al. [60] demonstrated an increase in the expression of pro-inflammatory cytokines, including TNF-α, in postmortem prefrontal cortex samples from depressed patients. In the present study, we observed a nearly significant DEX-induced increase in IL-6 and TNF-α levels in the HT also (Figure 4A and Figure 5A). Of note, corticosteroids were found to inhibit TNF-α synthesis under various inflammatory conditions through different mechanisms (e.g., suppression of the NF-κB pathway) [38]. Consistently, in the present study, 2 mg/kg DEX significantly decreased TNF-a levels in the HC of UCMS-subjected rats (Figure 4B). However, in contrast, our results show that TNF-α (and IL-6) levels were significantly elevated in the FC of DEX-treated UCMS rats.

The controversies regarding the effects of corticosteroids on mood are well-documented. Some studies have shown that corticosteroids, including DEX [61], exhibit potent antidepressant effects in patients with major depression. Other studies reported that corticosteroid treatment (especially at high doses) may lead to depression and mania [41,42,43]. Thus, administration of DEX to manic patients may appear risky and provocative. We note, however, that there are many clinical-practice precedents in which a treatment that was initially categorized as an absolute contraindication for a specific illness subsequently became a mainstream and accepted treatment strategy (e.g., β-adrenergic blockers as a treatment for congestive heart failure [62]). This was the logic behind examining the efficacy of DEX in mania-like models in rats in the present study.

It seems that the net effect of DEX and other corticosteroids on mood is affected by the dose, treatment duration and route of administration of each compound and their influence on levels of pro-and anti-inflammatory mediators in various regions of the brain. Of course, the variability in patients’ responses to corticosteroids treatment also originates from the heterogeneity in the patients’ basic and clinical characteristics. It is worth noting that, in the present study, PGE2 levels were significantly increased in the HT and FC of rats that were subjected to the UCMS protocol (Figure 6A,C). Treatment with DEX significantly decreased PGE2 levels in the FC and HC and in the HT in particular (Figure 6). The DEX-associated reduction in brain PGE2 levels may explain its antidepressant effects under certain circumstances. Support for this assumption may come from the following: (i) expression of the enzymes COX-2 and membrane PGE synthase were found elevated in postmortem FC samples of patients with bipolar disorder as compared to control subjects [15]; and (ii) treatment with selective COX-2 inhibitors was found to be beneficial in randomized clinical trials of depressive patients [19]. Furthermore, we found that DEX did not mitigate the AMPH-induced increase in locomotor activity (Figure 2) nor reduce the aggressiveness of animals in the RIAT behavioral model (Figure 3). These findings suggest (under the experimental conditions of this study) that, though DEX did not exert anti-manic-like effects, it also did not induce pro-manic effects. This is important to emphasize because previous studies have shown that DEX has the potential to induce manic symptoms [63].

Our study has some limitations. First, similarly to other studies, we used behavioral models that do not fulfill all validity criteria for bipolar disorder [54,64]. We utilized the RIAT paradigm [54] in order to expand the scope of the behavioral experiments and include another facet of bipolar disorder: aggressiveness. Another limitation of the study is that we used PTF as a “TNF-α inhibitor” while, in actuality, this drug exerts multiple pharmacological effects that may have also contributed to its observed beneficial behavioral outcomes. However, as discussed above, most available selective anti-TNF-α compounds have kinetic shortcomings that restrict their relevance and use for the treatment of neuro-inflammatory and psychiatric conditions [37]. Thus, we utilized the BBB-penetrating property of PTF in order to conduct this proof-of-concept study. Furthermore, currently it is not understood why the UCMS protocol did not induce any change in TNF-α and IL-6 levels in the tested brain regions (Figure 4 and Figure 5, respectively) as compared to the increase in PGE2 levels in the HC and FC of UCMS-subjected rats (Figure 6). However, this finding was repeatedly obtained in our experiments in male rats. A similar study in our lab also revealed that the UCMS protocol did not alter TNF-a and IL-6 levels in male rats but profoundly decreased TNF-a levels in female rats (Rostevanov et al.; personal communication). Further research is necessary to investigate the effects of various stress protocols on brain inflammatory mediator levels and on the gender-associated differences of this effect.

## 4. Materials and Methods

### 4.1. Animals

Male Sprague–Dawley rats approximately seven weeks of age and weighing 200–220 g at the beginning of the experiments were used throughout the studies. Animals were kept under controlled environmental conditions: ambient temperature 22 ± 1 °C, humidity 55–60%, 12 h light: 12 h dark photoperiod cycle, fed Purina Lab chow and water ad libitum. Only healthy animals with no signs of sickness were included in the studies. All experiments complied with the ARRIVE guidelines and were carried out in agreement with the instructions of the Committee for the Use and Care of Laboratory Animals at Ben-Gurion University of the Negev, Israel (authorization number IL-08-09-2014).

### 4.2. Behavioral Studies

Rats were housed three per cage under controlled conditions (unless otherwise indicated). All behavioral studies were conducted during the dark phase. Before the initiation of behavioral studies, rats were adapted to the housing conditions for 4–7 days, after which they were subjected to the behavioral experiments.

#### 4.2.1. Open Field Test

This test was used to measure the locomotor activity of rats. The open field arena was made of a black box (60 cm (W) × 80 cm (L) × 60 cm (H)), which was divided into a 30% central zone and a 70% peripheral zone. Rats were placed in the corner of the arena. Sessions were video-taped by a camera placed approximately one meter above the center of the arena and subsequently assessed using a video-tracking system (Ethovision, XT 14; Noldus Information Technology, Wageningen, the Netherlands). A 5% ethanol in water solution was used to clean the arena before the introduction of each animal. The parameters that were analyzed for each session were total distance traveled, scalar mean velocity and time spent in the peripheral versus central zones of the arena [13,65]. An increase in the duration of time spent in the central zone, in comparison to control animals, was recognized as risk-taking behavior [13].

#### 4.2.2. UCMS Paradigm

The UCMS protocol consisted of chronic exposure to different mild stressors every day for certain periods of time, including: grouped housing (six instead of three rats per cage), placement in a tilted cage (30°, 3 h), food deprivation (18 h), water deprivation (18 h), placement in a filthy cage (8 h) and exposure to perfume odor (8 h) [55,66]. Every day of the UCMS protocol included 3–5 stressors overall, and at each time-point the animals were subjected to a maximum of two stressors.

#### 4.2.3. Porsolt’s FST

The FST was used to assess depressive-like behavior [54,55]. Rats were positioned inside a glass cylinder (100 cm (H), 40 cm (diameter)) filled midway with water (24–28 °C). Immobility time (representing despair and depressive-like behavior) was the measure that was determined in this test. The behavior of rats during FST sessions was videotaped and subsequently evaluated by an experienced observer who was blinded to the treatment protocol.

#### 4.2.4. AMPH-Induced Hyperactivity Test

This is a widely used animal model for assessing mania-like behavior [65]. Rats were given a single intraperitoneal (ip) injection of AMPH (0.5 mg/kg; D-amphetamine sulfate, Bulk 281, Bio-Techne Ltd., Abingdon, UK) to induce hyperactivity and risk taking behavior [65]. Then, rats were placed in a corner of an open field for a 20 min session, which was recorded and subsequently analyzed by a video-tracking system (Ethovision, Noldus, Wageningen, the Netherlands). The parameters that were measured were total distance traveled, mean velocity and time spent in the central zone of the arena.

#### 4.2.5. RIAT

In order to expand the scope of the investigation and avoid restricting the examination of DEX and PTF to a psychostimulant-induced hyperactivity model of mania, we utilized another behavioral test for mania: the RIAT, which models aggression, another facet of mania [54]. Similar to a previous protocol [54], we created “resident” (=aggressive) and “intruder” (=subject to aggression) animals. Creation of residents was done by individually housing 10–12 week old rats for 4 weeks (individual housing increases aggressiveness in rodents). Creation of intruders was done by group housing (three per cage, as usual) 6–8 week old rats for 4 weeks. Thus, intruder rats transitioned to adulthood by the end of group housing, yet they were still younger than the residents. During test sessions, resident rats were transferred in their home cage to a test room. After a five-minute adaptation period, an intruder rat was brought into the resident’s cage and behavior was monitored for 20 min. Sessions were video-taped and subsequently evaluated by an experienced observer who was blinded to treatment group delegation. The number of attacks by residents against intruders was the measure that was scored.

### 4.3. Treatment with Anti-Inflammatory Drugs

Either DEX (Teva Ltd., Petah Tikva, Israel, Lot 2571114) or PTF (Sigma-Aldrich, St. Louis, MO, USA, Catalog number P1784) were administered once daily for two weeks through an ip injection. The drugs were dissolved in a sterile 0.9% NaCl solution (vehicle). Each drug was given at two doses: DEX—1 or 2 mg/kg [67]; PTF—10 or 50 mg/kg [49,50]. It is important to mention that DEX and PTF themselves did not affect the normal locomotor activity of the animals, as determined in the open field test (data not shown).

### 4.4. Assessment of Depressive-like Behavior

**Figure 7:**
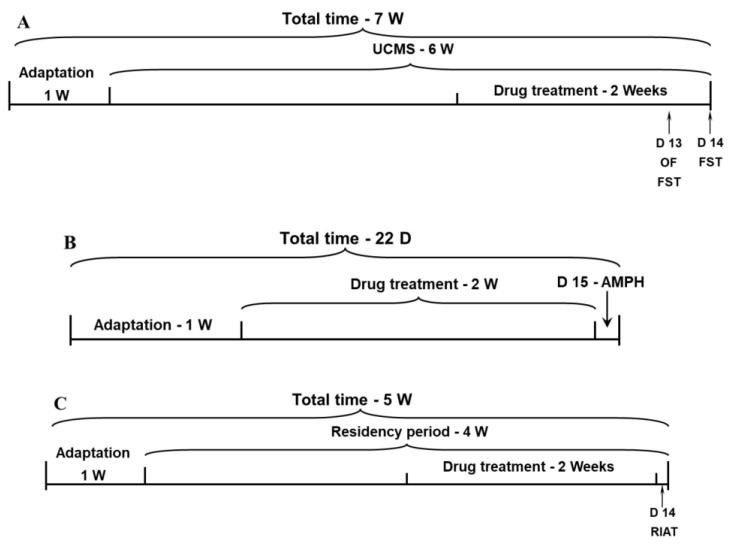
Timeline for the behavioral experiments. (**A**) Timeline of the protocol for the assessment of depressive-like behavior. (**B**) Timeline of the protocol for the assessment of mania-like behaviors. (**C**) Timeline of the protocol for the assessment of aggressive-like behavior. Abbreviations: AMPH, amphetamine; D, day; FST, forced swim test; OF, open field; RIAT, resident-intruder aggression test; UCMS, unpredictable chronic mild stress; W, week.

Rats were subjected to the UCMS protocol for six weeks, during the last two weeks of which animals were treated with DEX or PTF, as described above. On the morning of day 13 of the anti-inflammatory drug treatment, spontaneous locomotor activity was assessed in the open field test. In the afternoon, rats underwent a pretest 5 min session of FST. On day 14, rats were subjected to a 5-min FST test session. The timeline of this experiment is illustrated in Figure 7A.

### 4.5. Assessment of Mania-like Behaviors

(A) Rats were treated with DEX and PTF for 14 days. On day 15, rats were subjected to the AMPH-induced hyperactivity test, as described above. The timeline of this experiment is illustrated in Figure 7B. (B) To examine the effects of DEX and PTF on aggressive behavior, resident rats (see Section 4.2.5) were treated with each drug for the last two weeks of the “residency” period (four weeks). The RIAT was conducted on the last day of drug treatment. The timeline of this experiment is illustrated in Figure 7C.

### 4.6. Preparation of Brain Samples

Levels of IL-6, PGE2 and TNF-α were analyzed in specific brain regions—the hypothalamus (HT), frontal cortex (FC) and hippocampus (HC)—of rats that were subjected to the UCMS protocol as a model for depression. We focused on these three brain regions (HT, FC and HC) because they have been repeatedly linked to the pathophysiology of bipolar disorder [68] as well as other mental illnesses, and are associated with altered brain inflammation in some psychiatric patients [15,60]. Preparation of the brain samples was performed exactly as described previously [65].

### 4.7. Measurement of IL-6, PGE2 and TNF-α Levels

Levels of IL-6, PGE2 and TNF-α in brain samples (supernatants) were determined with specific ELISA kits according to the manufacturer’s protocols (R&D Systems, Minneapolis, MN, USA; catalog numbers DY506, SKGE004B and DY510, respectively). The detection limits of the assays were as follows: IL-6—125–8000 pg/mL, PGE2—39–2500 pg/mL, TNF-α—62.5–4000 pg/mL. Whenever the level of the tested constituent was less than the lower detection limit of the assay, results were marked as “undetectable” and given a value of zero.

### 4.8. Statistical Analysis and Presentation of Data

At the beginning, normality tests were conducted to find out whether the examined variable was distributed normally using the Shapiro–Wilk and D’Agostino–Pearson tests. For evaluation of between-group differences, we used a one-way ANOVA test (with the two-stage linear step-up procedure of Benjamini, Krieger and Yekutieli correction), followed by a two-tailed independent-samples *t* test. Values of *p* < 0.05 were considered statistically significant. Results are presented as means ± SEM for the sample size, as indicated in each figure. Each figure represents the results of one out of two independent experiments demonstrating similar results. In total, 425 rats were used in all experiments. Results in Figure 4, Figure 5 and Figure 6 were calculated as follows: ELISA result (pg/mL) divided by sample weight in milligrams. Results are presented as pg/mg wet weight.

## 5. Conclusions

For the first time, the results of the present study suggest that PTF may be a potentially beneficial treatment for bipolar disorder, as it exerted both antidepressant-like and anti-manic-like effects. These therapeutic effects seem to derive at least partially from its abatement of pro-inflammatory mediator production in the brain. In contrast to PTF, despite being a potent anti-inflammatory drug, DEX did not exhibit beneficial behavioral effects under the experimental conditions of the study. Additional studies are warranted to investigate the molecular mechanisms that underlie the beneficial effects of pentoxifylline against mania-like and depression-like behavior in animals, including the mitogen-activated protein kinases and nuclear factor kappa B pathways.

## Data Availability

Data is contained within the article.
